# Correspondence

**Published:** 1821-06-01

**Authors:** 


### CORRESPONDENCE.

We duly received the two pamphlets, with the unknown author's letter, dated March 1, 1821. The subject will probably be taken into consideration at no distant period. Meanwhile the unknown writer is requested to accept the tender of our professional respect and esteem.

*Junius's* critique came to hand, and though we are compelled to acknowledge that most of the censures are just, yet they are too severe for a journal devoted to a science that ought to humanize the soul, subdue the angry passions, and harmonize the members of the profession. Junius must surely have seen that this has long been our object, and we are sorry that the caustic passages and general tenor of the critique disable us from making use of it. Had judgment been tempered with mercy, and all personal allusions avoided, we should have been happy to avail ourselves of the analytical and other critical portions of the paper. If Junius will commission any friend to call for the critique, stating in writing the motto, as voucher, it shall be returned.

---

### SCRIBLOMANIA.

In consequence of *Philo-Criticus's* letter, we have glanced over the productions to which he alludes, and find them to be mere compilations from the most common authorities, and indicative of no great talent in the authors, who appear, indeed, to have had little or no personal experience of the subjects on which they have undertaken to write. As some books must necessarily pass unnoticed, we think it best to leave those on one side which can yield nothing valuable to the public by the process of reviewing. Even with such precaution, this Journal has sometimes difficult and complicated functions to perform in its laboratory. Often has it recourse to the literary *pestle and mortar*, for the purpose of breaking down the crude materials preparatory to the *sifting* process. The alembic or retort is constantly at work to separate the *spirit* from the *caput mortuum* of medical productions. *Evaporation* also we find a very convenient process, where authors have dissolved their materials in such excessive quantities of verbal menstrua, (probably with the view of imitating certain mineral springs whose virtues depend on the diffusion of a small proportion of salts through a great volume of water,) that it requires considerable *tact* as well as *taste* to ascertain what is the impregnating ingredient. It not seldom happens, however, that we are able to arrive at our object by a much nearer route than evaporation—namely, by *precipitation.* A modicum of critical acumen, nay, of common sense, will frequently throw down, at once, the active principle from an immense infusion or solution; and we are thus enabled to present our readers with the *emetin or morphium* of an octavo, in a space not much exceeding that in which Hannibal carried his last anodyne draught. We need not dwell on the obvious and necessary processes of literary filtration, clarification, despumation, sublimation, digestion, extraction, &c. to which we have daily recourse; but we think it proper, for the honour of the profession, not to descend to those low *culinary* operations, of *pickling*, *peppering*, *roasting*, *and basting*, which some of our medical *critics* have introduced, for the purpose of exciting depraved appetites. At the same time we are free to confess that several *authors* have lately set literary *fare* before the public, which almost sanctions the use of those *sauces* and other *provocatives* held forth for sale by the critical cooks. To us, however, it appears the wisest plan, as well as the most dignified, to make *silence* censure. Nor can we approve of the *barbarous* practice pursued by some modern critics, (which smacks strongly of the *chirurgo-tonsorial* age) namely, that of *shaving* authors, with razors, too, which Neptune's barber-general would be ashamed to apply to the face of any customer on crossing the line. Indeed, from the literary food which *some* authors and *some* critics venture to bring into the market, these venders must look upon the public customers as belonging to that species of *simple* animals which consists in a *stomach* only, and consequently which cannot be supposed to be very delicate in their appetites. It must be confessed, however, that in this age of literary epicures the most fastidious stomach may find provender to its taste—in the ample range extending from the ponderous monograph that embraces but a single topic, to the slender coup d'œil, that gives us, at one glance, the quintescence of all things that exist in the heavens above, the earth beneath, or the waters under the earth. We think, therefore, that Philo-Criticus has no reason to complain of the want of Genius in the medical writers of the present day. For our own parts, we are often amazed at the extent of their ingenuity! It has been said, that "*ex nihilo nihil fit.*" We deny it. A whole volume may easily be generated now *ex nihilo*, and that of such dainty workmanship that it may be fit to—"set before a king," and *honourably* rewarded with knighthood for its *manu*facturer.*

---

*Extract of a Letter from Dr. Dickson.*

"As to the remarks of the Editor of the Intelligencer on my case of erysipelas, to say the best of them, they are founded on error; for were I as willing to find fault as he seems to be, I could point out three mistakes in as many lines. The patient did *not* require bleeding at my visit of the 26th; for the symptoms of the inflammation of the brain did not supervene until the night afterwards: from that period the disease was treated with equal vigour and success, and therefore your opinion was perfectly correct. With regard to my ordering ℥vi. of blood to be taken on the 25th, the case expressly states the 25th to have been "the day before I saw him." Such criticism is unworthy of any reply."

---

* It is highly probable that Dr. Eady, of *parietal* celebrity, will soon receive the order of knighthood (*night*-hood) for his ingenious invention of *nocturnal lithography*, as a substitute for *literary* or professional fame. We understand also, that Dr. (soon *Sir Richard*) Reece is about to present, at the next Levee, a superb copy of his "Reecean Pandect of Medicine," published a few years ago, and now to be embellished with a highly finished portrait of Joanna Southcote, in a most interesting *parturient* posture, the Doctor himself in attendance to receive the first fruits of Johanna, and *gazette the health* of the long promised Shiloah.

